# Suppression of Brown Adipocyte Autophagy Improves Energy Metabolism by Regulating Mitochondrial Turnover

**DOI:** 10.3390/ijms20143520

**Published:** 2019-07-18

**Authors:** Donghwan Kim, Ji-Hye Kim, Young-Ho Kang, Je Seong Kim, Sung-Cheol Yun, Sang-Wook Kang, Youngsup Song

**Affiliations:** 1 Department of Biomedical Sciences, University of Ulsan College of Medicine, Seoul 05505, Korea; 2 Bio-Medical Institute of Technology (BMIT), University of Ulsan College of Medicine, Seoul 05505, Korea; 3 Department of Clinical Epidemiology and Biostatistics, University of Ulsan College of Medicine, Seoul 05505, Korea; 4 Asan Institute for Life Sciences, Asan Medical Center, Seoul 05505, Korea

**Keywords:** autophagy, brown adipose tissues, mitophagy, energy homeostasis, aging

## Abstract

The high abundance of mitochondria and the expression of mitochondrial uncoupling protein 1 (UCP1) confer upon brown adipose tissue (BAT) the unique capacity to convert chemical energy into heat at the expense of ATP synthesis. It was long believed that BAT is present only in infants, and so, it was not considered as a potential therapeutic target for metabolic syndrome; however, the discovery of metabolically active BAT in adult humans has re-stimulated interest in the contributions of BAT metabolic regulation and dysfunction to health and disease. Here we demonstrate that brown adipocyte autophagy plays a critical role in the regulation BAT activity and systemic energy metabolism. Mice deficient in brown adipocyte autophagy due to BAT-specific deletion of *Atg7*—a gene essential for autophagosome generation—maintained higher mitochondrial content due to suppression of mitochondrial clearance and exhibited improved insulin sensitivity and energy metabolism. Autophagy was upregulated in BAT of older mice compared to younger mice, suggesting its involvement in the age-dependent decline of BAT activity and metabolic rate. These findings suggest that brown adipocyte autophagy plays a crucial role in metabolism and that targeting this pathway may be a potential therapeutic strategy for metabolic syndrome.

## 1. Introduction

The maintenance of systemic energy homeostasis involves the precise sensing of energy levels, integration and transduction of metabolic signals, and co-ordinated regulation of energy intake and expenditure. Dysregulation of any one of these processes may cause metabolic disorders. As a primary calorie reservoir and endocrine organ, the adipose tissue plays a central role in maintaining energy balance. In mammals, adipose tissues are classified into three distinct types, white adipose tissue (WAT), brown adipose tissue (BAT) and beige adipose tissue, according to anatomical location, morphology, and function. WAT is the major site of energy storage. Under conditions of over-nutrition, WAT preserves surplus energy as unilocular lipid droplets in the form of triglycerides, which are mobilized by lipolysis and delivered to other organs via the systemic circulation under conditions of energy depletion, to maintain metabolic homeostasis. Unlike WAT, BAT contains lipid droplets in multilocular forms and instead of storing energy, it uses glucose and lipid as fuel to generate heat and regulate thermogenesis [1]. Uncoupling protein 1 (UCP1) is the critical molecule conferring thermogenic activity to BAT. Whereas other tissues use the proton gradient generated from mitochondrial respiration for ATP synthesis, UCP1 specifically expressed in the mitochondrial inner membrane of BAT generates heat by allowing the ATP synthesis-independent (uncoupled) translocation of protons to the mitochondrial matrix [2,3,4,5]. BAT is characterized by rich mitochondrial content and dense vascularization, to facilitate heat transmission throughout the body [6,7,8]. Recently, a third adipocyte phenotype—beige adipocytes—was identified as interspersed throughout the subcutaneous WAT. Upon stimulation by a variety of signals—including chronic cold exposure, adrenergic signaling and exercise—these cells acquire BAT-like characteristics, while withdrawal of these stimuli induces reversion to a WAT-like phenotype.

Adipose tissue is one of the most metabolically active organs and it exhibits constant remodeling in response to changes in metabolic status through the synthesis and degradation of cellular components and organelles. One of the most important mechanisms regulating tissue remodeling is autophagy. Autophagy is a cellular catabolic process that wraps aberrant or superfluous cellular components within double-membrane vesicles termed autophagosomes, which are delivered to lysosomes for the degradation and recycling of macromolecules to be used in the synthesis of new cellular components [9]. In contrast to proteasomes—which are specialized for protein quality control—autophagy regulates the turnover of a wide range of cellular components, including proteins, lipids, and entire organelles, in the process of tissue remodeling [10,11].

Tissue-specific autophagy-related gene knockout mice have revealed unexpected functions for autophagy in major metabolic tissues. In the hypothalamus, autophagy is induced by starvation, and regulates the expression of AgRP (Agouti-related protein) and food intake [12]. Autophagy regulates the projection of anorexigenic pro-opiomelanocortin (POMC) neurons, and mice lacking POMC neuron autophagy exhibit increased food intake and decreased energy expenditure [13,14]. In the liver, autophagy regulates endoplasmic reticulum (ER) stress, and hepatic overexpression of the autophagosome biogenesis regulator gene *Atg7* improves obesity-induced steatosis and glucose metabolism [15]. Genetic ablation of *Atg7*—specifically in pancreatic β-cells—reduces total β-cell mass and insulin secretion [16,17], whereas *Atg7* deletion in the skeletal muscle improves whole-body energy and glucose metabolism by enhancing *Fgf21* expression [18]. Recently, Martinez-Lopez et al. (2013) reported that autophagy is involved in brown adipocyte differentiation, as knockout of *Atg7* in mouse Myf5^+^ lineages—common progenitor cells for brown adipocytes and skeletal myocytes—exhibited defective BAT and skeletal muscle development [19]. While these studies demonstrate critical roles for autophagy in the metabolism, autophagic function in mature brown adipocytes has not been addressed. Thus, in this study, we investigated the role of autophagy in brown adipocytes and whole-body energy homeostasis.

## 2. Results

### 2.1. Generation of Brown Adipocyte-Specific Atg7 Knockout Mice

**Figure 1:**
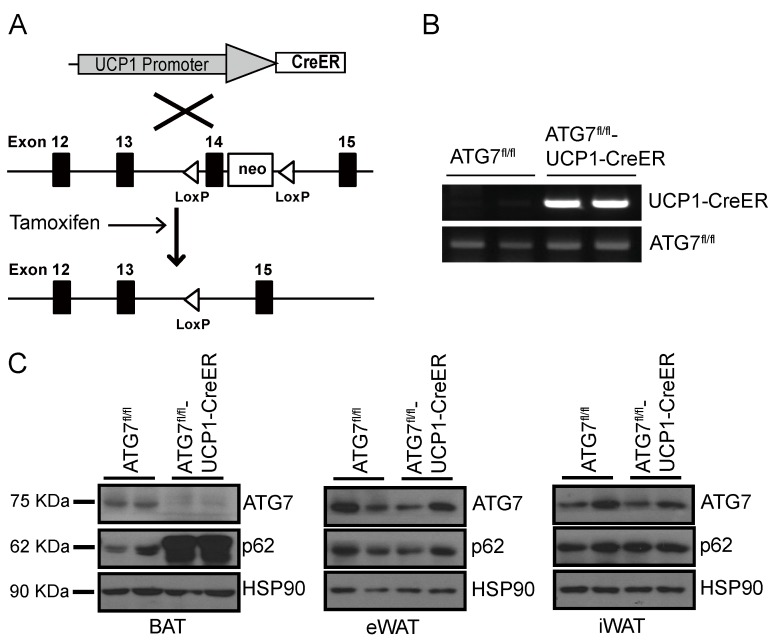
Generation of brown adipocyte-specific *Atg7* knockout (ATG7B KO) mice. (**A**). Schematic illustration of the strategy for establishing brown adipocyte-specific conditional autophagy-deficient mice. *Atg7*^fl/fl^ mice were crossed with uncoupling protein 1 (UCP1-CreER^+/^^−^) transgenic mice, and tamoxifen was administered to induce the nuclear migration of CreER. (**B**). Genotyping results for *Atg7*^fl/fl^ (control) mice and *Atg7*^fl/fl^-UCP1-CreER^+/^^−^ (ATG7B KO) mice. The top panel shows the genotyping results for the UCP1-CreER allele and the bottom panel shows the genotyping results for the *Atg7*^fl/fl^ allele. (**C**). Western blot analysis of ATG7 and p62 expression in brown adipose tissue (BAT) (left panel), epididymal white adipose tissue (eWAT; middle panel) and inguinal white adipose tissue (iWAT; right panel) of control and ATG7B KO mice.

The protein autophagy related 7 (ATG7) is an essential regulator of autophagosome formation, and multiple in vitro and in vivo studies have demonstrated that the genetic ablation of *Atg7* inactivates autophagy. To investigate the role of autophagy in brown adipocytes, we crossed *Atg7*-floxed mice (*Atg7*^fl/fl^) [20] with transgenic mice expressing the tamoxifen-dependent Cre recombinase (CreER) driven by the UCP1 promoter [21,22], and generated a brown adipocyte-specific autophagy-defective mouse model (*Atg*7^fl/fl^-UCP1-CreER^+/^^−^) (Figure 1a,b). Although the brown adipocytes of *Atg7*^fl/fl^-UCP1-CreER^+/^^−^ mice constitutively express CreER, recombination does not occur until CreER is activated by tamoxifen treatment. Compared to *Atg7*^fl/fl^ mice (referred to as control mice hereafter), oral administration of tamoxifen dramatically decreased Atg7 expression in BATs of *Atg7*^fl/fl^-UCP1-CreER^+/^^−^ mice (referred to as ATG7B KO mice hereafter) (Figure 1c, left panel). Conversely, Atg7 expression in epididymal WAT (eWAT) and inguinal WAT (iWAT) of ATG7B KO mice was comparable to that in control mice (Figure 1c, middle and right panels), confirming BAT-specific expression. Moreover, the autophagy marker protein p62 was upregulated only in BAT of ATG7B KO mice (Figure 1c). These results suggest that *Atg7* was specifically deleted in the BAT of ATG7B KO mice and thus inhibited autophagy exclusively in the BAT.

### 2.2. Suppression of BAT Autophagy Reduces Body Weight and Improves Glucose Metabolism

**Figure 2:**
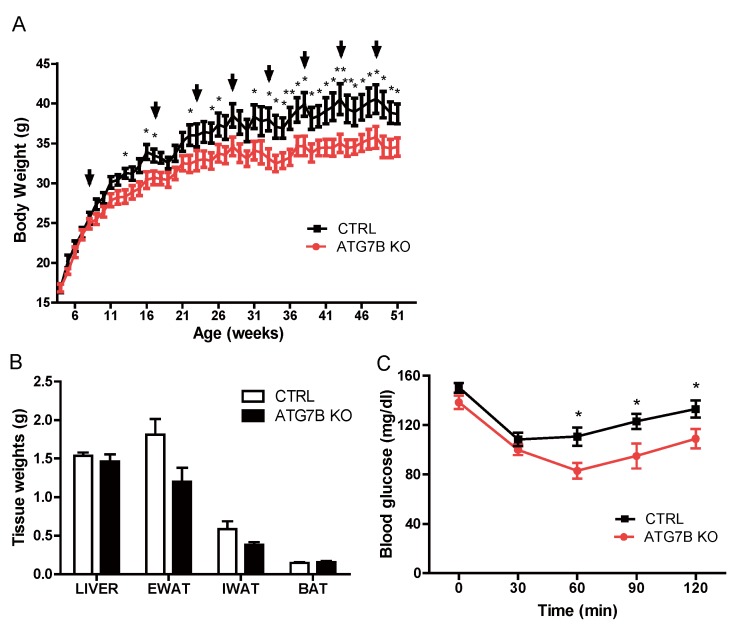
Brown adipocyte-specific *Atg7* knockout mice exhibit reduced body weight and improved insulin sensitivity. (**A**). Weekly body weight chart of control (*n* = 7) and ATG7B KO mice (*n* = 7), maintained on a normal chow diet. Arrows indicate weeks that control and ATG7B KO mice were treated with tamoxifen. (**B**). Weights of liver, eWAT, iWAT, and BAT from 1-year-old control (*n* = 7) and ATG7B KO mice (*n* = 7). (**C**). Insulin tolerance test (ITT) of normal chow-fed control (*n* = 7) and ATG7B KO mice (*n* = 7). Statistical significance was determined by Mann–Whitney test. * and ** represent *p* < 0.05 and *p* < 0.01 respectively.

After confirming the suppression of autophagy in BAT of ATG7B KO mice, we examined the impact of this brown adipocyte-specific autophagy deficit on metabolism. Initially, control and ATG7B KO mice displayed comparable body weights; however, tamoxifen administration suppressed body weight gain in ATG7B KO mice compared to control mice. The body weight difference between groups diverged progressively during the 5 months of tamoxifen treatment and was maintained thereafter (Figure 2a). At the age of 1 year—45 weeks after the initiation of tamoxifen administration—we dissected various organs from ATG7B KO and control mice and measured their weights. The weights of major metabolic tissues including liver, BAT, and WAT were comparable between ATG7B KO and control mice (Figure 2b). Consistent with improved metabolic efficiency, ATG7B KO mice exhibited greater whole-body insulin sensitivity as assessed by insulin tolerance testing (ITT) (Figure 2c).

### 2.3. Body Weight Reduction in ATG7B KO Mice Is Due to Enhanced Energy Expenditure

**Figure 3:**
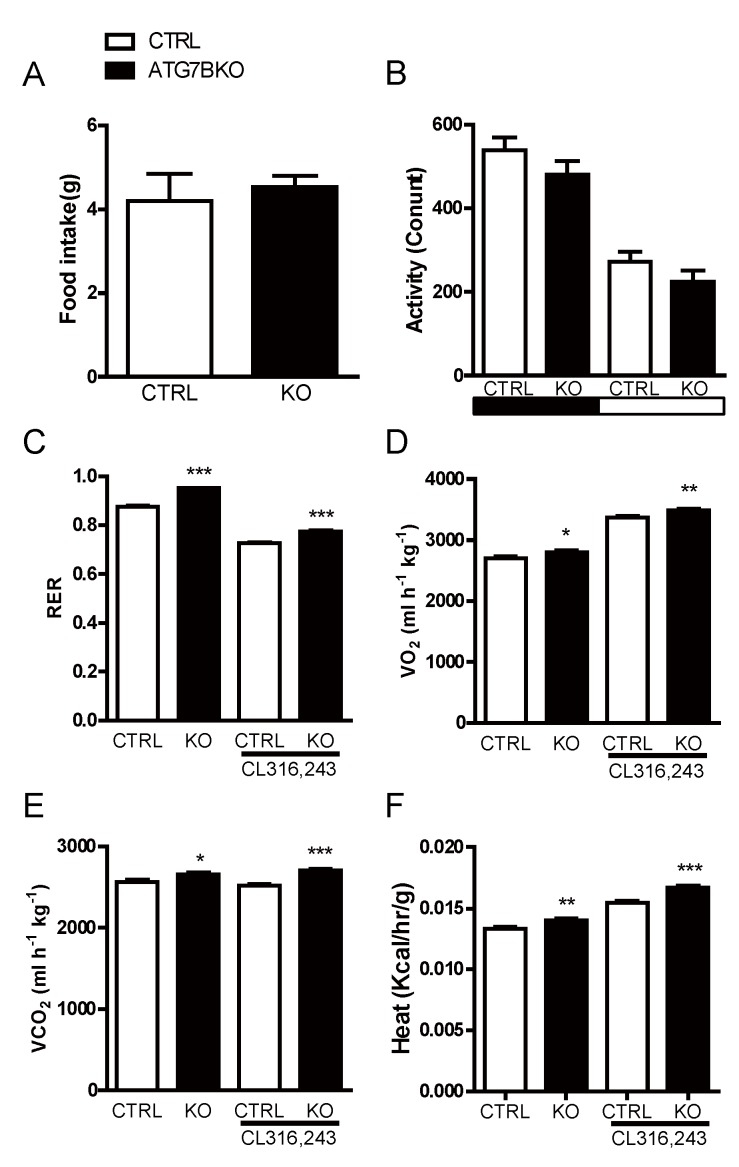
Increased energy expenditure in ATG7B KO mice. Shown are comparisons of (**A**). 24-h food intake and (**B**). physical activity of control (*n* = 3) and ATG7B KO mice (*n* = 5) (**C**). Respiratory exchange ratio (RER), (**D**). relative oxygen consumption, (**E**). carbon dioxide production, and (**F**). energy expenditure of night-time basal and CL316,243 treated control (*n* = 4) and ATG7B KO mice (*n* = 4), maintained on a normal chow diet were analyzed using indirect calorimetry. Statistical significance was determined by the Mann–Whitney test. *, ** and *** represent *p* < 0.5, *p* < 0.01 and *p* < 0.001 respectively.

In principle, reduced body weight can result from a decrease in energy intake, an increase in energy expenditure, or a combination of both. ATG7B KO mice—despite maintaining lower body weight (Supplementary Figure S1)—showed nearly identical 24-h food intake and physical activity relative to control mice (Figure 3a,b). We then examined if the body weight reduction observed in ATG7B KO mice was due to an alteration in energy expenditure from BAT, by comparing control and ATG7B KO mice in metabolic cage studies. Consistent with the improved insulin sensitivity (Figure 2c), the respiratory quotient (respiratory exchange ratio [RER]) was higher in ATG7B KO mice than control mice (Figure 3c). In addition, oxygen consumption (O_2_) and carbon dioxide production (CO_2_) rates in both basal and β3-AR stimulated conditions were substantially elevated in ATG7B KO mice (Figure 3d,e). As a result, energy expenditure was also higher in ATG7B KO mice than control mice (Figure 3f).

### 2.4. Increased Mitochondrial Content in BAT of ATG7B KO Mice

**Figure 4:**
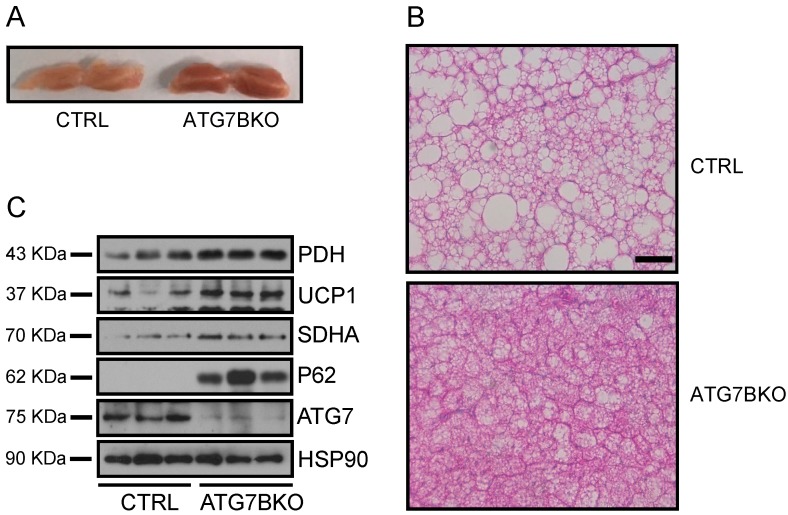
Increased mitochondrial content in ATG7B KO mice. (**A**). Appearance of BAT depots dissected from control and ATG7B KO mice. (**B**). Histological analysis of hematoxylin and eosin (H & E) stained sections of BAT from control and ATG7B KO mice. The scale bar represents 200 μm. (**C**). Immunoblotting for UCP1 and mitochondria-resident proteins PDH and SDHA in BAT depots of control and ATG7B KO mice.

The BAT of ATG7B KO mice was redder and contained fewer and smaller lipid vacuoles than the BAT of control mice (Figure 4a,b). Brown adipocyte lipid content is associated with the rate of fatty acid oxidation—which occurs in the mitochondrial matrix—and mitochondrial content and activity are often proportional to brown adipocyte activity. These relationships suggest elevated mitochondrial content in the BAT of ATG7B KO mice. Indeed, mitochondrial content—as assessed by the expression levels of the mitochondria-localized proteins [23,24] pyruvate dehydrogenase (PDH), succinate dehydrogenase complex flavoprotein subunit A (SDHA), and UCP1—was upregulated in the BAT of ATG7B KO mice compared to controls (Figure 4c). Conversely, mitochondrial protein expression levels were comparable in the eWAT and iWAT of ATG7B KO and control mice, suggesting that increased mitochondrial content was specific to the BAT (Supplementary Figure S2).

**Figure 5:**
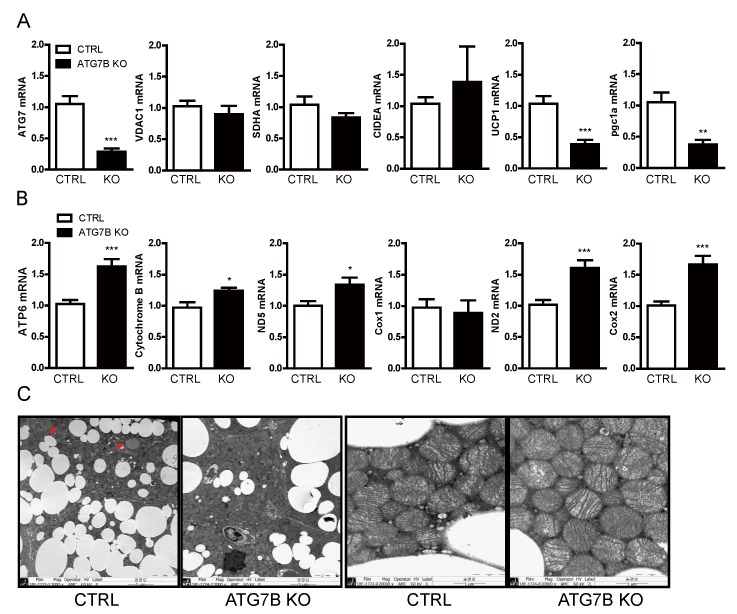
Suppression of mitochondrial turnover in BAT of ATG7B KO mice. Relative mRNA levels of (**A**). ATG7, PGC1α and the chromosome-encoded mitochondrial proteins VDAC1, SDHA and CIDEA, and (**B**). the mitochondrial-encoded proteins ATP6, cytochrome b, ND2, ND5, Cox1, and Cox2 from BAT of control and ATG7B KO mice as quantified by quantitative reverse PCR (qRT-PCR). Statistical significance was determined by Mann–Whitney test. *, ** and *** represent *p* < 0.05, *p* < 0.01, and *p* < 0.001 respectively. (**C**). Transmission electron microscopy images of the BAT from control and ATG7B KO mice. Left two panels (3000×), right two panels (20,000×).

To decipher the mechanisms regulating mitochondrial content by brown adipocyte autophagy, we first examined the mRNA levels of genes encoding mitochondrial proteins. Contrary to the upregulation of SDHA and UCP1 protein levels in BAT of ATG7B KO mice (Figure 4c), SDHA mRNA level was nearly identical and UCP1 mRNA level was actually lower in ATG7B KO mice compared to control mice. Further, the mRNA levels of two other chromosome-derived mitochondrial proteins—voltage-dependent anion channels (VDAC1) and cell death-inducing DFFA-like effector a (CIDEA)—were also comparable in the BAT of ATG7B KO and control mice (Figure 5a). In contrast, the mRNA levels of the mitochondria-encoded genes ATP6, CytB, ND2, ND5 and Cox2 were higher in ATG7B KO mice than in control mice (Figure 5b). Moreover, the mRNA level of Pgc1α—a critical player in mitochondrial biogenesis [25]—was significantly lower in the BAT of ATG7B KO mice, suggesting that the increase in the BAT mitochondrial content of ATG7B KO mice is caused by the inhibition of mitochondrial turnover rather than enhanced mitochondrial biogenesis. Transmission electron microscopy showed fewer lipid vacuoles in the BAT of ATG7B KO mice, but comparable mitochondrial appearance relative to control mice (Figure 5c), suggesting no difference in mitochondrial quality.

### 2.5. Age-Associated Increase in Brown Adipocyte Autophagy

**Figure 6:**
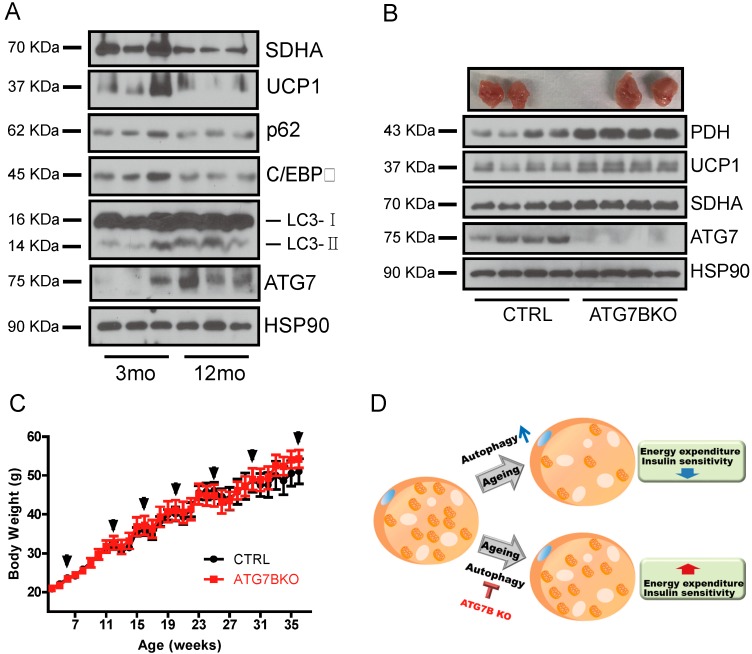
Age-associated changes of BAT autophagy and mitochondrial content. (**A**). The relative protein levels of mitochondrial (SDHA, UCP1) and autophagy (ATG7, p62) markers in BAT of young (3-month-old) and aged (12-month-old) mice (**B**). Appearance of BAT depots and immunoblotting results for ATG7, UCP1 and mitochondrial-resident proteins from BAT of control and ATG7B KO mice maintained on a 60% high-fat diet (HFD). (**C**). Body weight chart of control (*n* = 7) and ATG7B KO mice (*n* = 9) fed 60% HFD. Arrows indicate weeks that control and ATG7B KO mice were treated with tamoxifen. (**D**). Schematic illustration proposing the role of brown adipocyte autophagy in age-associated decline of BAT activity.

BAT mass and activity are often negatively correlated with age and body weight, and the chronic downregulation of BAT activity results in BAT whitening and eventual degeneration [26,27]. We directly examined if reduced BAT mass in aged and obese mice is associated with autophagy. Consistent with previous findings, the total amount of mitochondrial protein was lower in BAT of older mice compared to young mice. Older mice also maintained higher levels of autophagy proteins ATG7 and LC3-2 in BAT than younger mice, suggesting that diminished BAT activity with age may be associated with decreased mitochondrial content due to enhanced autophagy (Figure 6a). Finally, to test whether the suppression of BAT autophagy attenuates diet-induced obesity, we challenged control and ATG7B KO mice with a 60% high-fat diet (HFD). Consistent with the reduced autophagic elimination of mitochondria, ATG7B KO mice maintained elevated levels of mitochondrial protein expression in BAT (Figure 6b and Supplementary Figure S3a). However, diet-induced BAT whitening and body weight gain were comparable in both groups, so ATG7B KO alone failed to prevent diet-induced obesity (Figure 6c and Supplementary Figure S3b).

## 3. Discussion

The unique expression of UCP1 allows brown adipocytes to expend chemical energy as heat, via the uncoupling of mitochondrial respiration from ATP synthesis. Thus, in contrast to WAT—which serves as a storage depot for excess energy—the primary function of BAT is to maintain body temperature. While numerous pharmacological and genetic studies in rodent models have demonstrated the critical role of BAT in metabolic regulation [28,29,30,31], it was the discovery of BAT in adult humans that re-ignited interest in the therapeutic potential of BAT activation for the treatment of metabolic syndrome. Adult human BAT is present in the supraclavicular, neck, and paraspinal regions, and its activity is negatively correlated with body mass index (BMI), obesity, diabetes, environmental temperature, and age [26,32,33,34,35,36]. Total BAT activity depends on the rate of fatty acid oxidation, UCP1 expression and activity, brown adipocyte number, and mitochondrial content [37]. Thus, increases in any or all of these factors can increase the total thermogenic activity of BAT.

Here, we investigated if autophagy can regulate brown adipocyte activity by modulating these aforementioned factors. Indeed, we showed that brown adipocyte-specific *Atg7* knockout mice displayed improved BAT activity and whole-body energy metabolism due to increased mitochondrial content and UCP1 expression (i.e., greater thermogenic capacity). Mitochondrial content is regulated dynamically by the rate of mitochondrial biogenesis and turnover. Peroxisome proliferator-activated receptor gamma coactivator-1α (Pgc1α) is a transcriptional coactivator that acts as the master regulator of BAT mitochondrial biogenesis and thermogenic activity, by activating the transcription of mitochondrial transcription factor A (Tfam) and UCP1 [38,39]. Adipose tissue-specific Pgc1α and Tfam knockout mice develop insulin resistance associated with reduced mitochondrial content [40,41]. On the other hand, excess or damaged mitochondria are cleared through selective autophagy, termed mitophagy [42,43,44]. Our data suggest that increased mitochondrial content in the BAT of ATG7B KO mice results from the inhibition of mitochondrial clearance (mitophagy). Unexpectedly, despite increased mitochondrial content, the transcript levels of Pgc1α and UCP were downregulated in BAT of ATG7B KO mice. Although we have no direct evidence, we suspect that autophagy deficiency may activate a negative feedback mechanism to suppress mitochondrial biogenesis signaling and thermogenic activity. Future research is warranted to elucidate the molecular mechanisms through which biogenic and clearance systems communicate to regulate mitochondrial homeostasis.

Whereas autophagy has unique effects on metabolism in different tissues [12,13,14,15,16,17,18,45,46,47], it appears that the major function of autophagy in adipose tissue is the regulation of mitochondrial homeostasis [48]. Intriguingly—in line with previous observations in cardiac and skeletal muscle [49,50]—we found that the expression levels of mitochondrial markers were lower in the BAT of older mice than younger mice. Moreover, while p62 expression was downregulated, the expression levels of ATG7 and LC3-2 were elevated in BAT of older mice, suggesting that the age-dependent decline of BAT activity [51,52]–and the increased sensitivity to hypothermia with aging [34,53]—might be due to hyperactive mitophagy. On the other hand, while previous studies have observed upregulated autophagic activity in adipose tissue under obesity and diabetes [54,55,56,57] and the suppression of diet-induced obesity by the inhibition of adipose tissue autophagy [58,59,60,61], ATG7B KO failed to prevent diet-induce obesity despite maintaining elevated mitochondrial content. Thus, inhibition of brown adipocyte autophagy alone is not sufficient to protect from diet-induced obesity. Recently, the Kajimura group demonstrated that the β3-adrenergic receptor (β3-AR) agonist treatment of UCP1^+^ adipocyte-specific autophagy-null mice enhanced beige adipocyte maintenance and attenuated diet-induced obesity [61]. While their study discovered the importance of beige adipocytes in systemic energy homeostasis, their model system might contain some potential limitations. First, since β3-adrenergic signaling is also associated with the maintenance and recruitment of classical brown adipocytes, it cannot confirm whether the metabolic phenotypes observed in their mouse model are solely due to enhanced beige adipocyte maintenance. Second, because UCP1 is constantly expressed not only in mature adipocytes but also during the differentiation of BAT, Cre-mediated autophagy suppression could influence BAT development [62,63]. Conversely, although we adopted an inducible CreER system and tried to focus on the autophagy function in mature brown adipocytes, because a substantial amount of UCP1 is expressed in subcutaneous WAT (i.e., beige adipocytes in iWAT),. Tthere is a possibility that the improved metabolic phenotypes of ATG7B KO mice could also be due to beige adipocyte autophagy suppression. The development of more specific brown or beige adipocyte markers will clarify the significance and contributions of brown and beige adipocytes to whole-body energy homeostasis.

In summary, the current study demonstrates that brown adipocyte autophagy plays a critical role in the regulation of BAT activity and whole-body energy metabolism. However, while focusing on these, we failed to investigate the influence of brown adipocyte autophagy suppression on different components of metabolic syndrome [64]. For example, BAT is a rich source of vascular endothelial growth factor (VEGF), fibroblast growth factor 21 (FGF21), and adenosine, which are critical players in determining blood pressure via regulating vascularization and vasodilation [65,66,67]. Furthermore, there is a good correlation between body weight and blood pressure [68,69,70], and the age-dependent decline of BAT activity has been observed in hypertensive rats [71]. Therefore, it would be interesting to examine whether ATG7B KO mice maintain lower blood pressure and whether targeting brown adipocyte autophagy may protect against age-associated metabolic syndrome.

## 4. Materials and Methods

### 4.1. Animals

All animal experiments were performed according to an approved protocol (approval number: 2016-12-139, approved date: 9 August 2016), from the Institutional Animal Care and Use Committee of Asan Life Science Institute, Asan Medical Center, Seoul, Korea. Mice were housed in a temperature-controlled pathogen-free facility under a 12 h/12 h light/dark cycle (lights on at 08:00) with free access to water and a normal chow diet (Purina Rodent Chow, Seoul, Korea). To establish brown adipocyte-specific *Atg7* conditional knockout mice, Atg*7*^fl/fl^ mice [20,46] were crossed with UCP1-CreER^+/^^−^ mice [21]. Tamoxifen (1 mg/5 g body weight) (Sigma-Aldrich, St. Louis, MO, USA) dissolved in corn oil (Sigma-Aldrich) was orally administered to their off-spring for five consecutive days. To ablate *Atg7* from newly generated brown adipocytes, every fifth week tamoxifen was regularly administered to mice for five consecutive days [21]. In this experiment, *Atg7*^fl/fl^ mice and *Atg7*^fl/fl^-UCP1-CreER^+/^^−^ mice (ATG7B KO) were used as the control and experimental groups, respectively, and their body weights were monitored every week for 1 year. For diet-induced obesity—1 week after tamoxifen administration—the normal chow diet was switched to a 60% HFD (D12492, Research Diets, New Brunswick, NJ, USA).

### 4.2. Indirect Mouse Calorimetry Study

For acclimation, control and ATG7B KO mice were individually housed two days prior to the metabolic cage study. The oxygen (O_2)_ consumption, carbon dioxide (CO_2)_ production, locomotor activity, respiratory exchange ratio (RER) and food intake of individually housed mice were monitored using an indirect calorimeter (Columbus Instruments, Columbus, OH, USA) and analyzed as described previously [47]. For β3-adrenergic receptor stimulation experiments, 100 μg/g of CL316,243 (Sigma-Aldrich) was intraperitoneally injected.

### 4.3. Insulin Tolerance Test

For the ITT, 1 unit/kg of insulin (Humulin, Lilly, Indianapolis, IN, USA) was intraperitoneally administered to 4 h fasted control and ATG7B KO mice, and glucose levels in blood collected from a tail vein were monitored with an Accu-Chek Performa glucometer (Roche, Basel, Switzerland) every 30 min for 2 h.

### 4.4. Histology

Briefly, eWAT, iWAT, and BAT isolated from control and ATG7B KO mice were immediately fixed with 4% paraformaldehyde for between 1 and 2 days and embedded in paraffin.Sections were prepared at 5 µm thickness and stained with hematoxylin and eosin. For transmission electron microscopy (Jeol Ltd., Tokyo, Japan) analysis, isolated BAT was fixed in 2.5% glutaraldehyde solution in 0.1 M phosphate buffer overnight and then for 90 min with 1% osmium tetroxide before further processing.

### 4.5. mRNA Analysis

Isolated BAT was immediately frozen in liquid nitrogen and kept at −80 °C for further processing. Total RNA was isolated from BAT ground in liquid nitrogen using the RNA Mini Kit (Favorgen, Ping-Tung, Taiwan) according to the manufacturer’s instructions [72]. First-strand cDNA was synthesized with 550 ng of isolated total RNA and random hexamer using reverse transcriptase (Toyobo, Osaka, Japan) and mRNA expression was analyzed by real-time quantitative RT-PCR using a Light cycler480 (Roche, Basel, Switzerland) with Thunderbird SYBR green qPCR mix (Toyobo). Primers used for the amplification of specific genes are listed in the Supplementary Table S1. The expression of mRNA was normalized to that of L32.

### 4.6. Antibodies and Immunoblots

Snap-frozen mouse tissues were ground in liquid nitrogen and lysed with lysis buffer (20 mM HEPES pH 7.5, 200 mM NaCl, 1 mM EDTA, 1 mM EGTA, 0.5% Triton X-100, 20 mM Na-pyrophosphate, 50 mM β-glycerophosphate and 50 mM NaF) containing a protease inhibitor cocktail (Tech and Innovation, Chuncheon, Korea). Antibodies against ATG7, p62, SDHA, PDH, LC3 (Cell Signaling Technology, Danvers, MA, USA), C/EBP (Santa Cruz Biotechnology, Inc., Santa Cruz, CA, USA) and UCP1 (Abcam, Cambridge, UK) were used for immunoblotting analysis as indicated in the figures. Expression of HSP90 (Santa Cruz Biotechnology, Inc.) was measured as a gel loading control.

### 4.7. Statistics

All data are expressed as mean ± S.E.M. Group means and were compared by the nonparametric Mann–Whitney test using GraphPad Prism software (Available online: www.graphad.com). *p* < 0.05, *p* < 0.01, and *p* < 0.001 are represented by *, **, and ***, respectively, and they were considered statistically significant.

## Supplementary Materials

Supplementary materials can be found at https://www.mdpi.com/1422-0067/20/14/3520/s1.

## Abbreviations

BAT	brown adipose tissue
eWAT	epididymal white adipose tissue
iWAT	inguinal white adipose tissue
UCP1	uncoupling protein 1
Atg7	autophagy related 7
RER	respiratory exchange ratio
KO	knock out
