# Supplementary material for: Suppression of Brown Adipocyte Autophagy Improves Energy Metabolism by Regulating Mitochondrial Turnover
Source: Int J Mol Sci. 2019 Jul 18;20(14):3520. doi: 10.3390/ijms20143520 (PMC6678363; doi:10.3390/ijms20143520)
Supplement: Supplementary file 1 [file ijms-20-03520-s001.pdf]

Supplementary Materials

Supplementary Table S1. List of primers for quantitative reverse transcription-PCR (qRT-PCR).

Gene	Forward primer	Reverse primer
L32	5'-AGATCCTGATGCCCAACATC-3'	5'-CAGCTCCTTGACATTGTGGA-3'
ATG7	5'-GCTGGTACCTTGGGGTGTAA-3'	5'-CACGGGATTGGAGTAGGAGA-3'
VDAC1	5'-TTCGTCATTCTCGCCGAACA-3'	5'-CCAACCCTCATAGCCAAGCA-3'
SDHA	5'-ACCCAGACCTGGTGGAGACC-3'	5'-GGATGGGCTTGGAGTAATCA-3'
CIDEA	5'-CTCGGCTGTCTCAATGTCAA-3'	5'-TCCTTAACACGGCCTTGAAC-3'
UCP1	5'-GGATTGGCCTCTACGACTCA-3'	5'-TGCCACACCTCCAGTCATTA-3'
Pgc1α	5'-CCGAGAATTCATGGAGCAAT -3'	5'-TTTCTGTGGGTTTGGTGTGA -3'
ATP6	5'-GGCACCTTCACCAAAATCAC-3'	5'-CGGTTGTTGATTAGGCGTTT-3'
Cytochrome B	5'-TTTTATCTGCATCTGAGTTTAATCCTGT-3'	5'-CCACTTCATCTTACCATTTATTATCGC-3'
ND2	5'-GCCTGGAATTCAGCCTACTAGC-3'	5'-GGCTGTTGCTTGTGTGACGA-3'
ND5	5'-AGCATTCGGAAGCATCTTTG-3'	5'-TTGTGAGGACTGGAATGCTG-3'
Cox1	5'-GCCTTTCAGGAATACCACGA-3'	5'-AGGTTGGTTCCTCGAATGTG-3'
Cox2	5'-ACCTGGTGAACTACGACTGCT-3'	5'-CCTAGGGAGGGGACTGCTCA-3'

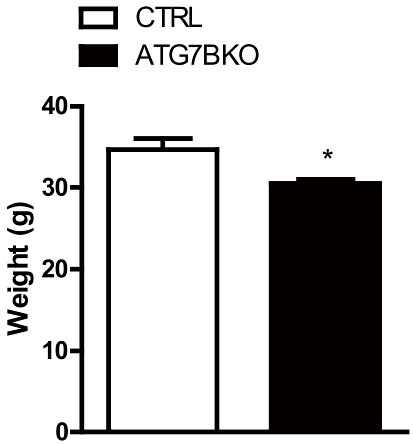

Supplementary Figure S1. Body weights of control and ATG7B KO mice maintained on normal chow diet (*n* = 3–5).

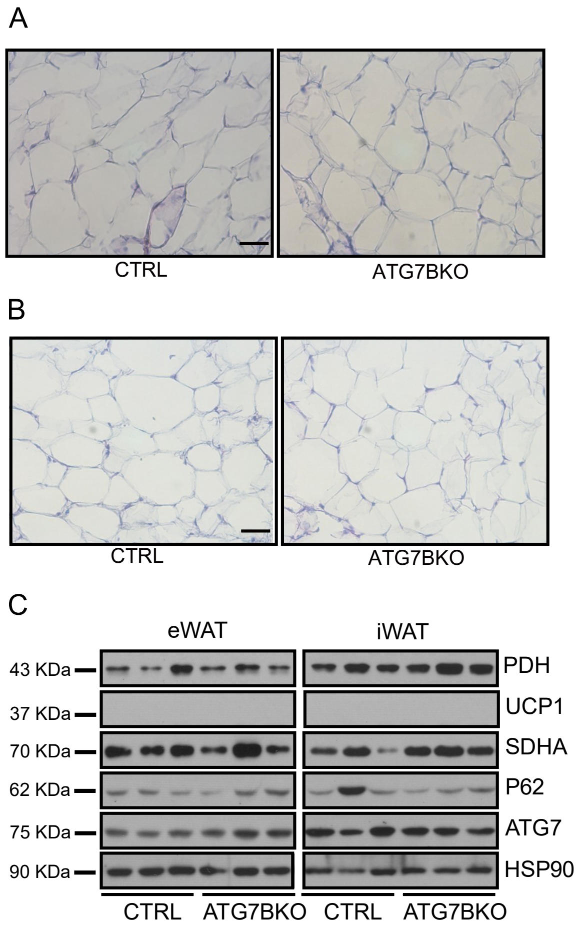

Supplementary Figure S2. Microscopic images of hematoxylin and eosin-stained sections prepared from A. epididymal white adipose tissue (eWAT) and B. inguinal white adipose tissue (iWAT) of control and ATG7B KO mice maintained on normal chow diet. Scale bar represents 200 μm. C. Immunoblotting for uncoupling protein 1 (UCP1) and mitochondria-resident proteins pyruvate dehydrogenase (PDH) and succinate dehydrogenase complex flavoprotein subunit A (SDHA) in eWAT (left panel) and iWAT (right panel) depots of control and ATG7B KO mice fed normal chow diet.

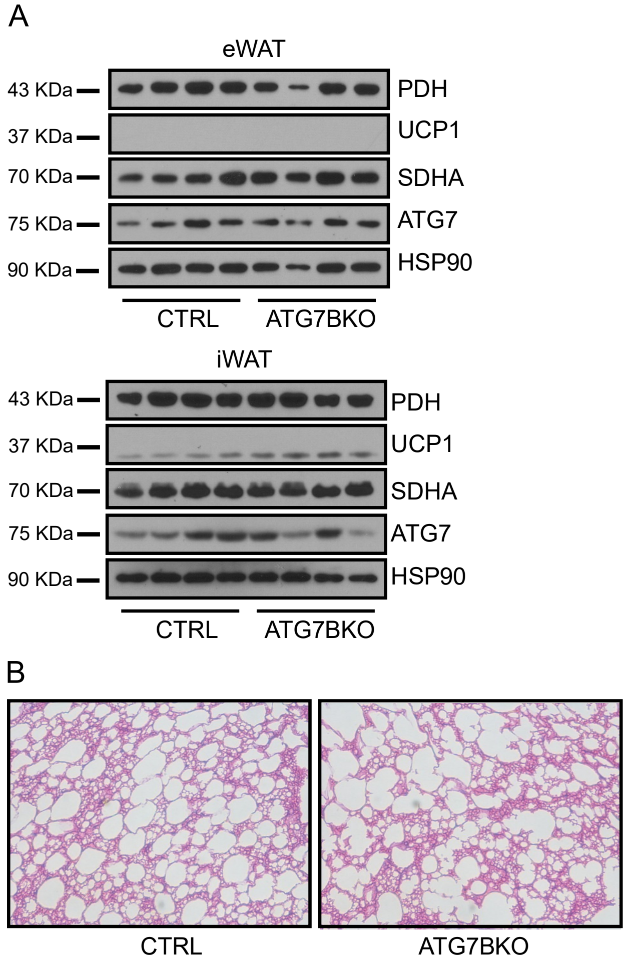

Supplementary Figure S3. A. Immunoblotting for ATG7, uncoupling protein 1 (UCP1), pyruvate dehydrogenase (PDH), and succinate dehydrogenase complex flavoprotein subunit A (SDHA) in epididymal white adipose tissue (eWAT, upper panel) and inguinal white adipose tissue (iWAT, lower panel) depots of control and ATG7B KO mice fed 60% high-fat diet (HFD). B. Histological analysis of hematoxylin and eosin-stained sections of brown adipose tissue (BAT) from 60% HFD-fed control and ATG7B KO mice.
